# ASO Author Reflections: Improved Access, Improved Outcomes: Regional Increases of Curative-Intent Liver Therapies Are Associated with Improved Survival in Patients with CRCLM

**DOI:** 10.1245/s10434-025-17605-1

**Published:** 2025-06-02

**Authors:** Vasileios Tsagkalidis, Brett L. Ecker

**Affiliations:** 1 Division of Surgical Oncology, Banner MD Anderson Cancer Center, Gilbert, AZ USA; 2 Division of Surgical Oncology, Rutgers Cancer Institute, New Brunswick, NJ USA; 3 Rutgers Robert Wood Johnson Medical School, New Brunswick, NJ USA; 4 Cooperman Barnabas Medical Center, Livingston, NJ USA

## Past

Colorectal cancer is the second leading cause of cancer death in the USA.^1^ Liver-directed therapies—including hepatectomy, ablation, and transplantation—are the cornerstone of curative treatment for patients with colorectal cancer liver metastases (CRCLM). There are no randomized data to confirm the survival benefit of these interventions compared with systemic therapy alone, with prior studies largely relying on patient-level observational data and subject to inherent limitations of selection bias and other unmeasured confounders.^2–4^ Thus, the precise impact of liver-directed therapies on survival remains incompletely defined.

## Present

Area-level analyses can be used to estimate the effect of an intervention when random assignment is not feasible. The current study leveraged the observed variations in liver therapy rates across health service areas (HSAs) from 163 HSAs in the linked SEER-Medicare database to assess the precise impact of such changes on survival.^5^ This method allows each area to serve as its own control while adjusting for observed changes in the patient population over time. Given that approximately one fifth of resection patients demonstrate long-term survival, we hypothesized that each 5% rise in the regional rate of interventions would lead to a 1% increase in population-level survival. In accordance, we observed that each 5% increase in the rate of liver therapies within an HSA was associated with a statistically significant 1.2% (95% confidence interval (CI) 0.4–2.0%) increase in risk-adjusted survival. Hence, modest increases in use of these interventions across a population can yield a measurable impact on regional CRCLM survival.

## Future

Our results confirm the survival benefit associated with surgical resection and quantify the expected impact of policies and interventions that increase utilization of liver-directed therapies. This underscores the need for system-level efforts to expand access to liver-directed therapies for this population. Future work will need to identify modifiable barriers (e.g., geographic and provider-level) to both surgical referral and subsequent intervention, as well as the factors contributing to the variability in liver therapy rates across different health service areas as a potential source of disparity in cancer outcomes.
